# The Impact of the COVID-19 Pandemic on Depression and Sexual Function: Are Pregnant Women Affected More Adversely?

**DOI:** 10.1055/s-0041-1736174

**Published:** 2021-11-16

**Authors:** Ramazan Denizli, Önder Sakin, Kazibe Koyuncu, Nayif Çiçekli, Nihat Farisoğulları, Mikail Özdemir

**Affiliations:** 1 Department of Gynecology, Arhavi State Hospital, Artvin, Turkey; 2 Department of Gynecology, Kartal Dr. Lutfi Kirdar City Hospital, Istanbul, Turkey; 3 Department of Gynecology, Mus State Hospital, Mus, Turkey; 4 Department of Gynecology, Viransehir State Hospital, Sanliurfa, Turkey; 5 Department of Public Health, Marmara University School of Medicine, Istanbul, Turkey

**Keywords:** COVID-19, pandemic, pregnant, depression, sexual function, Covid-19, pandemia, grávida, depressão, função sexual

## Abstract

**Objective** To investigate depression and sexual function among pregnant and non-pregnant women throughout the COVID-19 pandemic.

**Methods** A total of 188 women, 96 pregnant and 92 non-pregnant were included. The Beck Depression Inventory (BDI) and the Arizona Sexual Experience Scale (ASEX) were applied to the participants after obtaining sociodemographic data.

**Results** The depression scores of pregnant and non-pregnant women were similar ( *p* = 0.846). We found that the depression scores were significantly higher among the group of participants who have lower economic status ( *p* = 0.046). Moreover, the depression score was significantly higher among women who lost their income during the pandemic ( *p* = 0.027). The score on the ASEX was significantly higher, and sexual dysfunction was more prevalent among women who have lower levels of schooling and income ( *p* < 0.05). Likewise, the ASEX scores were significantly higher ( *p* = 0.019) among the group who experienced greater income loss throughout the pandemic. Upon comparing the pregnant and non-pregnant groups, we detected that sexual dysfunction had a significantly higher rate among pregnant women ( *p* < 0.001).

**Conclusion** In times of global crisis, such as the current pandemic, low-income families have an increased risk of experiencing depression and sexual dysfunction. When we compared pregnant women with non-pregnant women, depression scores were similar, but pregnant women were at a 6.2 times higher risk of developing sexual dysfunction.

## Resumo

**Objetivo** Investigar a depressão e as funções sexuais de mulheres grávidas e não grávidas durante a pandemia de Covid-19.

**Métodos** Um total de 188 mulheres, 96 grávidas e 92 não grávidas, foram incluídas. O Inventário de Depressão de Beck (Beck Depression Inventory, BDI, em inglês) e a Escala de Experiências Sexuais do Arizona (Arizona Sexual Experience Scale, ASEX, em inglês) foram aplicados aos participantes após a obtenção dos dados sociodemográficos.

**Resultados** As pontuações de depressão de mulheres grávidas e não grávidas foram semelhantes ( *p* = 0,846). Verificou-se que as pontuações de depressão foram significativamente maiores no grupo de participantes de menor nível econômico ( *p* = 0,046). Além disso, a pontuação de depressão foi significativamente maior em mulheres que perderam sua renda durante a pandemia ( *p* = 0,027). A pontuação na ASEX foi significativamente maior, e a disfunção sexual foi mais prevalente em pessoas com menores escolaridade e nível de renda ( *p* < 0,05). Da mesma forma, as pontuações na ASEX foram significativamente mais altas ( *p* = 0,019) no grupo que experimentou maior perda de renda durante a pandemia. Ao comparar os grupos de gestantes e não gestantes, detectou-se que a disfunção sexual apresentava índice significativamente maior entre as gestantes ( *p* <0,001).

**Conclusão** Em tempos de crise global, como a atual pandemia, famílias de baixa renda têm um risco maior de sofrer depressão e disfunção sexual. Quando comparamos mulheres grávidas e mulheres não grávidas, as pontuações de depressão foram semelhantes, mas as mulheres grávidas apresentaram um risco 6,2 vezes maior de desenvolver disfunção sexual.

## Introduction

Caused by the severe acute respiratory syndrome coronavirus 2 (SARS-CoV2), coronavirus disease 2019 (COVID-19) began with the first suspicious cases in November 2019 in Wuhan, Hubei, China, in late December. After that, the disease shortly spread throughout the world, in about three months. It was declared a pandemic by the World Health Organization (WHO) on March 12, 2020, and the first case in Turkey was reported on March 11, 2020. 1 2 The entire world is facing an unprecedented crisis due to the rapidness, depth, and scope of the pandemic. The physical, mental, and social well-being of people is being adversely affected throughout the pandemic, and the national and international measures taken against it have directly or indirectly affected people's economic well-being. Movement restrictions, one of the measures to slow down the spread of the pandemic, negatively affected the supply of labor. As has been the case in the global market, Turkey has also experienced an increase in the unemployment rate and decreased incomes. 3 A decrease in people's economic welfare levels in society can increase the rates of depression. 4

Previous studies 5 6 have revealed that the tendency to develop depression among females is higher than among males. Some studies 6 7 performed during the pandemic found that females were affected more adversely compared with the males. The mother might experience emotional fluctuations during pregnancy due to the anxiety she feels for both herself and the fetus. 8 The physiological and psychosocial changes that occur during pregnancy increase the tendency of developing depression and anxiety. In this regard, pregnant and new mothers are included in the vulnerable and high-risk group for depression. 9 10

The female sexual response cycle can be divided into four phases: baseline, arousal (excitement), orgasm, and resolution. Regarding these phases, women might experience various sexual dysfunctions, such as lack of sexual drive and arousal, and inability to orgasm during sexual activity. It is an underestimated problem with a general prevalence between 20% and 50%. 11 On the other hand, it has been demonstrated that a significant decrease in sexual activities occurs with pregnancy. Physical discomfort, fear of injuring the fetus, loss of sexual drive, physical awkwardness, painful sexual activity, and perceived lack of attraction are among the reported causes of this decrease in sexual activity during pregnancy. 12

The present research aimed to investigate the depression and sexual functions of pregnant and non-pregnant women throughout the COVID-19 pandemic.

## Methods

The present is a cross-sectional trial conducted from May to August, 2020 with 96 healthy pregnant and 92 nonpregnant women, totalling a sample of 188 women. Most of the pregnant and non-pregnant women whom we invited to the study did not want to participate; they wanted to be in the hospital for a shorter time because they were afraid of contact transmission by the doctor. In total, 188 healthy women aged between 18 and 45 years who sought care at the Gynecology and Obstetrics Outpatient Clinic of a hospital in Turkey, and who did not have any known disorder, had not received medication for chronic conditions, and voluntarily agreed to participate in our study were included. Pregnant women who had fetal or maternal problems during their pregnancy, women who had a medical history of psychiatric or sexual dysfunction, and participants who submitted incomplete questionnaires were excluded from the study. The participants read and signed the informed consent form; after that, we collected sociodemographic data from them, and applied the Beck Depression Inventory (BDI) and Arizona Sexual Experience Scale (ASEX).

Approval of the Ethics Committee was obtained (2020/514/176/9), with the project name of 2020–05–07T14_43_09, from the Ministry of Health of the Republic of Turkey to conduct the relevant scientific research.

Sociodemographic data, such as age, level of schooling, occupational status, and obstetric status was obtained from the patients. They were asked whether they were pregnant or not, and, if they were, their gestational week was requested, and they were divided into three groups based on the trimester: first trimester (up until the 14th gestational week), second trimester (14th to 28th gestational weeks), and third trimester (28th to 42th gestational weeks) groups. Based on data from May 2020 from the Turkish Statistical Institute, those with a total family monthly income below the hunger limit (2,438 Turkish Lira [TL]) are considered the lower-income class, those below the poverty line (7,942 TL), the middle-income class, and those with a higher income (> 7,942 TL), the upper-income class. Regarding loss of income during the pandemic, the participants were examined in 3 groups:, those who had no income loss, those who lost up to 50% of their income, and those who lost more than 50% of their income.

The BDI was developed to measure the risk of depression, the level of the depressive symptoms, and the fluctuation in the severity of depression among adults. It is a self-administered questionnaire developed by Beck et al. 13 , which consists of 21 items. The items of the scale are scored from 0 and 3 points. Higher scores indicate greater symptom severity. In those diagnosed with depression, scores of between 0 and 13 indicate minimal depression, from 14 to 19, mild depression, from 20 to 28, moderate depression and, from 29 to 63, severe depression. 14 The validity and reliability study of the Turkish adaptation of the questionnaire was performed by Hisli. 15

The Arizona Sexual Experience Scale (ASEX), which has been developed by McGahuey et al. 16 to briefly and efficiently screen and identify the problems experienced by patients in their sexual life, is a five-item rating scale that measures sexual drive, arousal, vaginal lubrication/penile erection, ability to have an orgasm, and satisfaction from orgasm; and it also has separate questionnaires specific to male and female subjects. We used the female form (questionnaire detailed to female patients) in the present study. The total score ranges from 5 to 30, and lower scores manifest that the sexual response is strong, easy, and satisfying, whereas higher scores display sexual dysfunction. The higher the scores, the more sexual dysfunction there is. 16 Based on the ASEX scores, the participants were included in the healthy group (scores from 0 to 10), the group with moderate sexual disorder (scores from 11 to 17), and the group with severe sexual disorder (scores ≥ 18). The validity and reliability of the Turkish adaptation of the ASEX have been tested among patients with renal failure. 17

For the categorical values, *p* -values were calculated using the Chi-squared test. For the continuous variables, the *p* -values were calculated using the Mann Whitney U test the and Kruskall Wallis test. Multivariable logistic regressions were used to calculate odds ratios (ORs) and 95% confidence intervals (95%CIs) for the associations between sexual dysfunction and depressive symptoms. Potential a priori confounders (such as, age, occupational status, and parity) were included in the models. All statistical analyses were performed using The Statistical Package for the Social Sciences (IBM SPSS Statistics for Windows, IBM Corp., Armonk, NY, United States) software, version 25.0.

## Results

The mean age of the sample was 30.1 ± 6.4 years. The demographics of the participants are presented in Table 1 .

**Table 1:** Demographics of the study participants

Age (years)		Mean (standard deviation): 30.1 (6.4)	Median (range): 29.0 (18.0–45.0)
		*n*	%
**Level of schooling**	Primary school graduate	23	12.2%
	High school graduate	68	36.2%
	University graduate or higher	97	51.6%
**Occupational status**	Unemployed	100	53.2%
	Working in the public sector	57	30.3%
	Working in the private sector	31	16.5%
**Level of income**	Low	17	9.0%
	Medium	110	58.5%
	High	61	32.4%
**Pregnancy**	Absent	92	48.9%
	Present	96	51.1%
**Trimester of pregnancy**	First	20	20.8%
	Second	58	60.4%
	Third	18	18.8%
**Parity**	Nulliparous	70	37.2%
	Multiparous	118	62.8%
**Type of delivery**	Nulliparous	70	37.2%
	Cesarean	69	36.7%
	Vaginal	49	26.1%
**Total**		188	100.0%

In the univariate analysis of the factors affecting the presence of depression among the participants, severe depression was significantly more prevalent among those who were unemployed compared with the other groups. Besides, as the level of income decreases, the percentage of mild or moderate depression increases significantly ( *p* = 0.046) ( Table 2 ).

**Table 2:** Factors impacting depression among the study participants

		Healthy		Mildly depressed		Moderately/severely depressed		
		*n*	%	*n*	%	*n*	%	*p* -value
**Level of schooling**	Primary school graduate	5	21.7%	8	34.8%	10	43.5%	0.115
	High school graduate	21	30.9%	33	48.5%	14	20.6%	
	University graduate or higher	37	38.1%	41	42.3%	19	19.6%	
**Occupational status**	Unemployed	25	25.0%	45	45.0%	30	30.0%	**0.003**
	Working in the public sector	30	52.6%	20	35.1%	7	12.3%	
	Working in the private sector	8	25.8%	17	54.8%	6	19.4%	
**Level of income**	Low	3	17.6%	7	41.2%	7	41.2%	**0.046**
	Medium	34	30.9%	47	42.7%	29	26.4%	
	High	26	42.6%	28	45.9%	7	11.5%	
**Loss of income during the pandemic**	No	46	40.7%	46	40.7%	21	18.6%	0.124
	≤ 50%	9	22.5%	19	47.5%	12	30.0%	
	> 50%	8	22.9%	17	48.6%	10	28.6%	
**Pregnancy**	Absent	29	31.5%	41	44.6%	22	23.9%	0.846
	Present	34	35.4%	41	42.7%	21	21.9%	
**Trimester of pregnancy**	First	10	50.0%	7	35.0%	3	15.0%	0.097
	Second	22	37.9%	25	43.1%	11	19.0%	
	Third	2	11.1%	9	50.0%	7	38.9%	
**Parity**	Nulliparous	24	34.3%	31	44.3%	15	21.4%	0.936
	Multiparous	39	33.1%	51	43.2%	28	23.7%	
**Type of delivery**	Cesarean	24	34.8%	29	42.0%	16	23.2%	0.986
	Vaginal	15	30.6%	22	44.9%	12	24.5%	
**Relative infected with COVID-19**	No	56	36.8%	65	42.8%	31	20.4%	0.087
	Yes	7	19.4%	17	47.2%	12	33.3%	

In the univariate analysis of the BDI scores of the participants, they were significantly higher among those who were unemployed (mean = 13.8) compared with those working in the public sector (mean = 9.5) ( *p* < 0.001). We also found that, as the level of economic income decreases, depression scores increase significantly ( *p* = 0.009). The BDI score (mean = 13.9) was significantly higher among the participants who lost their income during the pandemic compared with those who did not experienced income loss (mean = 11.4) ( *p* = 0.001). Furthermore, the BDI scores (mean = 14.0) of the patients whose relatives were diagnosed with COVID-19 was significantly higher than the scores of those whose relatives were not diagnosed ( *p* = 0.013) ( Table 3 ).

**Table 3:** Factors impacting the score of Beck Depression Inventory

		Mean	standard deviation	Median	First quartile	Third quartile	*p* -value
**Level of schooling**	Primary school graduate	15.4	7.8	14.0	10.0	19.0	0.080
	High school graduate	12.3	5.4	12.0	8.0	16.0	
	University graduate or higher	11.6	6.2	11.0	8.0	15.0	
**Occupational status**	Unemployed	13.8	6.2	13.0	9.5	18.0	< 0.001
	Working in the public sector	9.5	6.0	9.0	5.0	12.0	
	Working in the private sector	12.9	5.2	13.0	8.0	16.0	
**Level of income**	Low	15.3	4.8	17.0	13.0	18.0	0.009
	Medium	12.7	6.6	12.0	8.0	17.0	
	High	10.9	5.6	11.0	7.0	14.0	
**Loss of income during the pandemic**	No	11.4	6.4	11.0	7.0	14.0	0.001
	Yes	13.9	5.8	14.0	10.0	18.0	
**Pregnancy**	Absent	12.5	6.2	12.0	8.0	17.0	0.389
	Present	12.1	6.3	11.0	7.5	16.0	
**Trimester of pregnancy**	First	11.1	6.4	10.0	7.0	15.0	0.081
	Second	11.9	6.7	10.0	7.0	14.0	
	Third	14.1	4.7	14.5	11.0	17.0	
**Parity**	Nulliparous	12.4	6.3	11.0	8.0	14.0	0.654
	Multiparous	12.3	6.2	12.0	8.0	17.0	
**Type of delivery**	Cesarean	12.0	5.8	13.0	8.0	16.0	0.888
	Vaginal	12.7	6.8	12.0	8.0	18.0	
**Relative infected with COVID-19**	No	11.9	6.4	11.0	8.0	16.0	0.013
	Yes	14.0	5.3	15.0	11.5	18.0	

We found that the rate of severe sexual disorder among pregnant women was significantly higher than that of the non-pregnant women ( *p* = 0.006). On the other hand, the percentage of moderate sexual dysfunction was significantly higher among multiparous women ( *p* = 0.016). When the participants were categorized based on their depression status, moderate sexual dysfunction was determined to be high in patients with mild depressive symptoms, whereas the rate of severe sexual dysfunction was found to be high in those with severe depressive symptoms ( *p* = 0.008) ( Table 4 ).

**Table 4:** Factors impacting sexual dysfunction

		Normal		Moderate sexual dysfunction		Severe sexual dysfunction		
		*n*	%	*n*	%	*n*	%	*p* -value
**Level of schooling**	Primary school graduate	1	4.3%	14	60.9%	8	34.8%	0.145
	High school graduate	14	20.6%	40	58.8%	14	20.6%	
	University graduate or higher	24	24.7%	57	58.8%	16	16.5%	
**Occupational status**	Unemployed	14	14.0%	62	62.0%	24	24.0%	0.088
	Working in the public sector	14	24.6%	34	59.6%	9	15.8%	
	Working in the private sector	11	35.5%	15	48.4%	5	16.1%	
**Level of income**	Low	0	0.0%	12	70.6%	5	29.4%	0.101
	Medium	21	19.1%	66	60.0%	23	20.9%	
	High	18	29.5%	33	54.1%	10	16.4%	
**Loss of income during the pandemic**	No	29	25.7%	63	55.8%	21	18.6%	0.123
	Yes	10	13.3%	48	64.0%	17	22.7%	
**Pregnancy**	Absent	23	25.0%	59	64.1%	10	10.9%	**0.006**
	Present	16	16.7%	52	54.2%	28	29.2%	
**Trimester of pregnancy**	First	4	20.0%	12	60.0%	4	20.0%	0.579
	Second	11	19.0%	29	50.0%	18	31.0%	
	Third	1	5.6%	11	61.1%	6	33.3%	
**Parity**	Nulliparous	22	31.4%	34	48.6%	14	20.0%	**0.016**
	Multiparous	17	14.4%	77	65.3%	24	20.3%	
**Type of delivery**	Cesarean	14	20.3%	43	62.3%	12	17.4%	**0.018**
	Vaginal	3	6.1%	34	69.4%	12	24.5%	
**Relative infected with COVID-19**	No	32	21.1%	88	57.9%	32	21.1%	0.783
	Yes	7	19.4%	23	63.9%	6	16.7%	
**Depression**	Normal	20	31.7%	29	46.0%	14	22.2%	**0.008**
	Mildly depressed	15	18.3%	56	68.3%	11	13.4%	
	Moderately/severely depressed	4	9.3%	26	60.5%	13	30.2%	

According to the univariate analysis, ASEX score decreases significantly as the level of schooling increases. Moreover, the ASEX score was higher among those who were unemployed. As the level of economic income decreases, the ASEX score increases significantly. We found that as the loss of income rose during the COVID-19 pandemic, the ASEX score also rose significantly. In addition to that, as depressive symptoms increased, the ASEX score increased as well ( *p* = 0.005) ( Table 5 ).

**Table 5:** Factors impacting the Arizona Sexual Experience Scale

		Arizona Sexual Experience Scale					
		Mean	Standard deviation	Median	First quartile	Third quartile	*p* -value
**Level of schooling**	Primary school graduate	16.2	3.3	15.0	14.0	19.0	0.034
	High school graduate	14.4	4.1	14.0	12.0	17.0	
	University graduate or higher	13.8	3.6	14.0	11.0	17.0	
**Occupational status**	Unemployed	14.9	3.6	15.0	13.0	17.0	0.032
	Working in the public sector	13.8	3.9	13.0	11.0	15.0	
	Working in the private sector	13.4	4.1	14.0	10.0	16.0	
**Level of income**	Low	15.9	2.6	16.0	15.0	18.0	0.040
	Medium	14.4	3.9	14.0	12.0	17.0	
	High	13.7	3.9	14.0	10.0	15.0	
**Loss of income during pandemic**	No	13.8	3.8	14.0	10.0	16.0	0.019
	≤ 50%	14.6	4.1	15.0	13.0	16.0	
	> 50%	15.7	3.3	16.0	13.0	18.0	
**Pregnancy**	Absent	13.3	3.3	13.0	10.5	15.0	< 0.001
	Present	15.2	4.0	15.0	13.0	18.0	
**Trimester of pregnancy**	First	14.6	3.8	15.0	13.0	17.0	0.260
	Second	15.1	4.4	15.0	12.0	18.0	
	Third	16.4	2.7	17.0	15.0	19.0	
**Parity**	Nulliparous	13.8	4.1	14.0	10.0	17.0	0.064
	Multiparous	14.6	3.6	15.0	13.0	17.0	
**Type of delivery**	Nulliparous	13.8	4.1	14.0	10.0	17.0	0.098
	Cesarean	14.3	4.0	15.0	12.0	17.0	
	Vaginal	15.0	3.1	15.0	13.0	17.0	
**Relative infected with COVID-19**	No	14.3	3.8	14.0	12.0	17.0	0.888
	Yes	14.2	3.9	14.0	12.0	16.0	
**Depression**	Normal	13.7	4.4	14.0	10.0	17.0	0.005
	Mildly depressed	14.0	3.1	14.0	12.0	15.0	
	Moderately/severely depressed	15.8	3.8	16.0	14.0	18.0	

When the factors affecting sexual dysfunction in women are analyzed by the multivariate analysis, odds ratio increase was found 1.18 times (95% CI 1.07–1.31), corresponding to each unit increase of age. Compared with those without depressive symptoms, the risk of sexual dysfunction in mildly-depressed patients increased 4.31 times (95%CI: 1.57 to 11.82), while this rate was of 5.57 (95%CI: 1.42 to 21.81) among participants with moderate/severe depression. The risk of sexual dysfunction increased 6.40 times (95%CI: 2.09 to 19.54) among unemployed patients compared with those employed in the private sector. Moreover, there was an increase of 6.20 in the OR (95% CI 2.15–17.90) among pregnant women compared with non-pregnant women ( Table 6 ).

**Table 6:** Multivariate analysis of the factors impacting sexual dysfunction

	**B**	**SE**	**Wald test**	***p*** **-value**	**Odds Ratio**	**95% confidence interval**	
						**Lower**	**Upper**
**Age (years)**	0.173	0.051	11.598	0.001	1.189	1.076	1.313
**Healthy**			10.153	0.006			
**Mildly depressed**	1.462	0.514	8.084	0.004	4.317	1.575	11.829
**Moderately/severely depressed**	1.718	0.696	6.089	0.014	5.573	1.424	21.812
**Working in the private sector**			10.696	0.005			
**Unemployed**	1.857	0.569	10.634	0.001	6.402	2.098	19.543
**Working in the public sector**	1.217	0.635	3.670	0.055	3.377	.972	11.731
**Pregnant**	1.826	0.540	11.421	0.001	6.209	2.153	17.905
**Multiparous**	0.267	0.539	0.245	0.620	1.306	0.454	3.758
**Loss of income**	1.040	0.545	3.647	0.056	2.830	0.973	8.230

B = value; SE = standard error.

When the correlation analysis was performed between the ASEX and BDI scores regarding the pregnancy status, a slightly positive significant correlation was found among pregnant women (r = 0.365; *p* < 0.001).

## Discussion

The expectant mother may experience emotional fluctuations throughout pregnancy because she is concerned about herself and the fetus. 18 Depression, which may be experienced during pregnancy, may lead to maternal and fetal complications. 8 The stress experienced throughout the pregnancy might trigger preterm labor through the activation of the pituitary and adrenal axis, and is also a risk factor for gestational hypertension and preeclampsia. 8 19 Previous researches have reported that the prevalence of depression during pregnancy ranges from 12% to 36%. 20 In the present study, we found that 42.7% of pregnant women had mild symptoms of depression throughout the pandemic, whereas 21% had moderetely-severe symptoms of depression. Similarly, depression rates in non-pregnant women were found to be significantly higher than studies conducted before the pandemic. In studies of depression in female university students in Turkey prevalence of ranges from 9.2% to 35.2%, in other countries between 18.5% and 52.6% (14a–14c). 6 21 22 In the present study, we found that 44.6% of non-pregnant women had mild symptoms of depression throughout the pandemic, whereas 22% had moderate/severe symptoms. A study 23 performed in Turkey during the pandemic reported that it has a considerable impact on the levels of depression and anxiety of pregnant women. In a multi-center cross-sectional study 24 performed in China comparing the mental state of pregnant women before and after the declaration of the COVID-19 pandemic, the authors reported that pregnant women who were examined after the announcement of the COVID-19 outbreak had significantly higher levels of anxiety and symptoms of depression.

Previous studies 25 26 have suggested that individuals who are in the lower socioeconomic class have a higher rate of mental disorders such as depression compared with individuals in middle and upper socioeconomic classes. Upon reviewing the literature, we could not detect one study that revealed a correlation between depression and parameters such as economic status and income loss during the COVID-19 pandemic. In the present study, the depression scores were significantly higher among the group with lower economic status. Likewise, the BDI score was significantly higher among patients who lost their income during the pandemic. The depression scores were significantly higher among females whose relatives have been infected with COVID-19.

The present study has demonstrated that the depression scores increased among women during the pandemic, which is in line with reports in the literature. 15 16 In the present study, we found that pregnant and non-pregnant women are adversely affected in a similar manner; on the other hand, the study suggests that circumstances such as being in the low-income class, experiencing loss of income, and having relatives infected with COVID-19 may increase the risk of developing depression.

Sexual life is one of the factors that affect the general health status and quality of life of women. Sexual dysfunction is a multifactorial problem influenced by various biological, psychological, and environmental factors. 27 It has been suggested 28 that sexual dysfunction is a common problem that increases with age and impacts 30% to 50% of women. Women who have an ASEX score ≥ 11 are considered to have sexual dysfunction. In a study 29 comparing pregnant and non-pregnant women in Turkey before the pandemic, the authors found high ASEX scores in 55.6% of pregnant women and in 23.2% of non-pregnant women. In the present study the rate of sexual dysfunction during the pandemic was of 83.3% among pregnant women, and of 75% among non-pregnant women. This situation indicated that the sexual lives of all women were negatively affected throughout the pandemic. A significantly higher rate of sexual dysfunction was also detected among pregnant women in the present study, and the OR was 6.2, which is in line with previous studies. 29 30

It is well documented that the level of socioeconomic well-being impacts sexuality. Previous studies 31 32 have reported that sexual dysfunction was more prevalent among women with a lower economic status. Similarly, in the present study, sexual dysfunction was significantly higher among women in the lower-income class and among those who lost their income during the pandemic. The risk of sexual dysfunction among unemployed women is 6.40 times higher than that of employed women. One study 33 demonstrated that sexual dysfunction is less common among women with higher levels of schooling. Likewise, the results of the present study support the aforementioned finding as well the fact that, as the level of schooling increases, the prevalence of sexual dysfunction decreases significantly.

Previous studies 34 35 36 37 have suggested that the previous number of births and the type of delivery do not impact the sexual function. In the present study, similar results were found when the ASEX scores were analyzed.

It has been claimed 38 that people under restriction have a high tendency to develop depression and anxiety, and that sexual intercourse while under restriction is a protective factor against depression. One study 39 found that several women who were using short-acting reversible contraception (SARC) discontinued their contraceptive method during the pandemic but continued to engage in sexual activity and had unplanned pregnancies. The BDI and ASEX scores were also observed in parallel in the present study ( Tables 3 , 4 ), and The BDI scores were significantly higher among women with sexual dysfunction.

This study has some limitations; Firstly, it is a cross-sectional trial and the long-term effects are unknown. Secondly, the study compared slightly different women in the same period, not different periods when the same women were pregnant and not pregnant. Finally, the negative impact of the pandemic detected among the participants may have been milder than the impact felt by women in other parts of Turkey, since the district where the present study was performed was considered a low-risk area for infection by SARS-CoV2.

## Conclusion

In the present study, women who lost their income during the pandemic had higher rates of depression and sexual dysfunction. Upon comparing the pregnant and nonpregnant women, we found that the rates of depression were similarly higher in both groups than the before pandemic, whereas the rate of sexual dysfunction was 6.2 times (95%CI: 2.1 to 17.9) higher among pregnant women. Low-income families and women have an increased risk of experiencing depression and sexual dysfunction in times of global crises, such as a pandemic.
